# Real-Time Structural Health Monitoring of Reinforced Concrete Under Seismic Loading Using Dynamic OFDR

**DOI:** 10.3390/s25185818

**Published:** 2025-09-18

**Authors:** Jooyoung Lee, Hyoyoung Jung, Myoung Jin Kim, Young Ho Kim

**Affiliations:** Korea Photonics Technology Institute (KOPTI), Gwangju 61007, Republic of Korea; wajjy@kopti.re.kr (J.L.); spring@kopti.re.kr (H.J.); mjinkim@kopti.re.kr (M.J.K.)

**Keywords:** dynamic optical frequency domain reflectometry, distributed strain sensing, reinforced concrete column, structural health monitoring, seismic resonance tracking

## Abstract

This paper presents a compact dynamic optical frequency domain reflectometry (D-OFDR) platform enabling millimeter-scale, distributed strain sensing for real-time structural health monitoring (SHM) of reinforced concrete subjected to seismic loading. The proposed D-OFDR interrogator employs a dual-interferometer architecture: a main interferometer for strain sensing and an auxiliary interferometer for nonlinear frequency sweep compensation. Both signals are detected by photodetectors and digitized via a dual-channel FPGA-based DAQ board, enabling high-speed embedded signal processing. A dual-edge triggering scheme exploits both the up-chirp and down-chirp of a 50 Hz bidirectional sweep to achieve a 100 Hz interrogation rate without increasing the sweep speed. Laboratory validation tests on stainless steel cantilever beams showed sub-hertz frequency fidelity (an error of 0.09 Hz) relative to conventional strain gauges. Shake-table tests on a 2 m RC column under incremental seismic excitations (scaled 10–130%, peak acceleration 0.864 g) revealed distinct damage regimes. Distributed strain data and frequency-domain analysis revealed a clear frequency reduction from approximately 3.82 Hz to 1.48 Hz, signifying progressive stiffness degradation and structural yielding prior to visible cracking. These findings demonstrate that the bidirectional sweep-triggered D-OFDR method offers enhanced real-time monitoring capabilities, substantially outperforming traditional point sensors in the early and precise detection of seismic-induced structural damage.

## 1. Introduction

Recent seismic events have underscored the necessity for real-time structural health monitoring (SHM) systems capable of tracking the deformation and degradation of reinforced concrete members during ground motion. Owing to their limited tensile ductility, RC elements can experience hidden cracking and localized yielding well before macroscopic damage is visible. Quantitative and spatially resolved data captured in situ mesurements are therefore indispensable for rapid safety assessment and emergency response. Conventional inspection techniques, such as computer-vision crack detection and ultrasonic testing, rely on controlled environmental conditions or are labor-intensive, making them ill-suited to continuous monitoring [1,2]. Point sensors remain the prevailing option for uninterrupted data acquisition: electrical resistance strain gauges offer kilohertz-rate sampling but require extensive wiring and are susceptible to corrosion and electromagnetic interference [3], whereas fiber Bragg grating (FBG) sensors mitigate electromagnetic issues and allow multiplexing, yet each grating still represents a discrete measurement position, leaving un-instrumented zones between them [3,4].

Distributed fiber-optic sensing (DFOS) mitigates these shortcomings by transforming an entire optical fiber into a continuous strain transducer. Among DFOS modalities, optical frequency domain reflectometry (OFDR) exploits Rayleigh backscatter to achieve millimeter-level spatial resolution over tens of meters while retaining the EMI immunity and small footprint characteristic of fiber-optic sensors. Advances in swept-laser technology and embedded signal processing have elevated OFDR update rates to more than 100 Hz, sufficient to resolve the dominant vibration modes of typical civil structures [5,6,7]. Although laboratory studies have shown that Rayleigh-based DFOS can capture mode shapes and detect cracks missed by point sensors [7], validation under realistic seismic loading conditions remains limited.

Field-test results indicate strong potential for DFOS in critical infrastructure monitoring. In concrete dams, DFOS provides dense strain–temperature fields that reveal seepage and differential movements between concrete blocks [8]. In tunnels, it resolves convergence, ovalization, and localized cracking with geodetic validation [9]. At expansion joints of a long-span railway bridge, DFOS captured joint-strain spikes and reconstructed three-dimensional girder deformation, enabling rapid post-earthquake safety confirmation after M6.4 and M6.8 events [10]. In practice, most deployments to date have been static or non-seismic, spanning railway tracks [11], pipelines [12], fatigue-tested RC beams [13], and in situ load tests on building elements [14]. Analytical methods now infer crack widths and deflections directly from Rayleigh-based DFOS strain profiles [15]. Collectively, these applications measure deformation, strain–temperature fields, convergence and cracking, and rotational and joint motion, in situ and in real time, enabling actionable monitoring across critical infrastructure.

Despite significant progress in DFOS, no system has yet been demonstrated that tracks, in real time, the seismic response and damage evolution of RC members in the field. Past earthquakes show that sparse strong-motion networks and post-event inspections have often proven insufficient to deliver structure-specific, timely diagnostics for critical RC elements. Rapid collapses of the Cypress Viaduct (Loma Prieta, 1989) [16] and the Hanshin Expressway (Kobe, 1995) [17], as well as the CTV building in Christchurch (2011) [18] and numerous soft-story frames in Mexico City (2017) [19], occurred without real-time insight into evolving demands on columns, joints, and bearings. If distributed fiber-optic sensing had been installed along pier/column heights and around bearings or beam–column joints, spatially localized strain and slip anomalies might have triggered pre-emptive closures or evacuations. These gaps motivate dense, distributed measurements to complement strong-motion stations and provide actionable early warnings for RC components.

In this paper, we address this gap through the development of a compact and dynamic optical frequency domain reflectometry (D-OFDR) platform for real-time seismic monitoring of reinforced concrete structures. The proposed D-OFDR interrogator delivers 3 mm spatial resolution along 10 m of fiber and achieves an effective 100 Hz frame rate by employing a dual-interferometer architecture with a wavelength-swept laser covering 4 THz and synchronized dual-edge triggering on both the up-chirp and down-chirp of a 50 Hz bidirectional sweep. Rising-edge triggers are used for up-chirps and falling-edge triggers for down-chirps, enabling two acquisition channels to operate in an interleaved fashion. This approach achieves a twofold effective frame rate without requiring higher performance from the laser, DAQ, or electronics. Laboratory calibration on two stainless steel cantilevers, designed with resonance frequencies of 4 Hz and 20 Hz, demonstrated sub-hertz frequency fidelity (error within 0.09 Hz) relative to co-located strain gauges. The system was subsequently bonded to a 2 m tall RC column and exposed 13 times to amplitude-scaled replicas (10–130%, peak 0.864 g) of the 1940 El Centro record on a shake table. Distributed strain histories combined with short-time Fourier analysis revealed a stepwise reduction in the column’s fundamental frequency from 3.82 Hz at a peak ground acceleration (PGA) of 10% to 1.48 Hz at a PGA of 130%, delineating four distinct damage regimes before visible cracking appeared. These results highlight D-OFDR as a powerful fiber-optic-based tool for quantitative, real-time structural health assessment of RC infrastructure under earthquake loading.

## 2. Performance Test

### 2.1. D-OFDR Interrogator Architecture and Dual-Edge Timing Scheme

**Figure 1:**
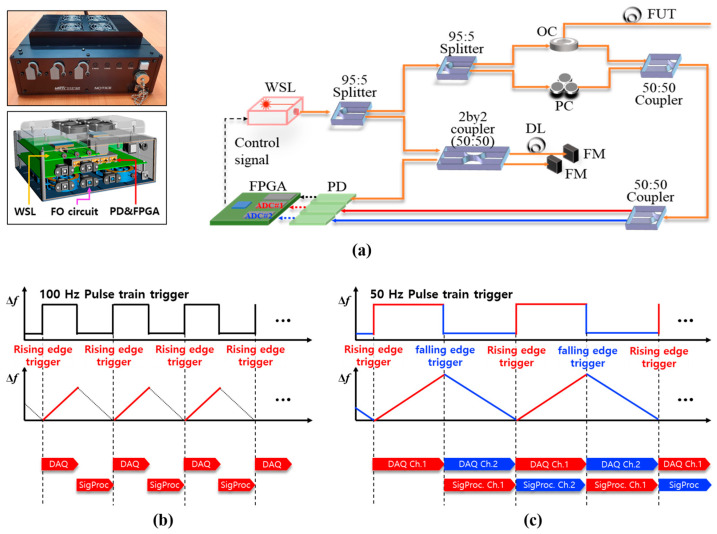
Dynamic OFDR interrogator: (**a**) D-OFDR hardware and fiber-opics schematic; (**b**) conventional single-edge timing for 100 Hz operation; (**c**) proposed dual-edge timing that uses both up-chirp (sweep) and down-chirp (retrace) triggers of a 50 Hz bidirectional sweep to achieve 100 Hz measurements. (FO: Fiber-optic, OC: optical circulator, FUT: fiber under test, DL: delay line, FM: faraday mirror, PD: photo-detector, FPGA: field programmable gate array).

Figure 1a shows the optical hardware layout of the compact D-OFDR system developed to measure the dynamic behavior of structures at a rate of 100 Hz. The optical circuit of the proposed system is composed of two interferometers: the main interferometer for strain sensing and the auxiliary interferometer for nonlinear frequency sweep compensation. Both interferometer signals are detected by photodetectors and sampled by a dual-channel data acquisition (DAQ) board that enables high-speed embedded signal processing on a field-programmable gate array (FPGA) allocated for sampled data of each channel. All optical and electronic components were integrated into a 250 mm × 250 mm × 110 mm aluminum enclosure weighing approximately 3.2 kg, facilitating single-operator installation. The wavelength-swept laser (WSL) board incorporates a tunable external-cavity diode that enables a triangular sweep from 1534 nm to 1566 nm, resulting in a total sweep range of 32 nm, which corresponds to an optical frequency range (∆f) of 4 THz. Considering a sweep rate (ν) set to 50 Hz and FUT length (L) was 10 m, the required frequency bandwidth (∆F) was approximately 19.5 MHz according to the following relation, ∆F=γτ, where γ denotes frequency sweep rate and τ denotes the optical delay time that equals to 2 *n*L/c. Here, *n* is the refractive index of optical fiber that corresponds to 1.46 and c is speed of light that corresponds to 3 × 10^8^ m/s.

For signal processing, WSL was configured to synchronize a sweep trigger signal for DAQ timing control. In a conventional OFDR system, as shown in Figure 1b, each frame is triggered solely at the start of either the up-chirp sweep or the down-chirp sweep, forcing data acquisition and signal processing to occur sequentially and thereby limiting throughput. In such a single-edge triggering scheme, a sweep rate of 100 Hz is required to complete both the up-chirp and down-chirp sweeps within a single interrogation cycle. In contrast, as shown in Figure 1c, the proposed system adopts a dual-edge triggering operation, which allows the sweep rate to be reduced by half without sacrificing interrogation rate. By issuing a rising-edge trigger for up-chirp and a falling-edge trigger for down-chirp, two independent acquisition channels operate in an interleaved fashion. While Channel 1 samples and processes the up-chirp, Channel 2 simultaneously handles the down-chirp, and vice versa in the next cycle. This parallelism aligns the data acquisition time with the embedded signal processing latency, enabling an effective interrogation rate of 100 Hz at a 50 Hz sweep rate and doubling the dynamic bandwidth without requiring higher performance from the WSL, the DAQ, frequency bandwidth, or other system components.

### 2.2. Real-Time Strain Measurement Test

**Figure 2:**
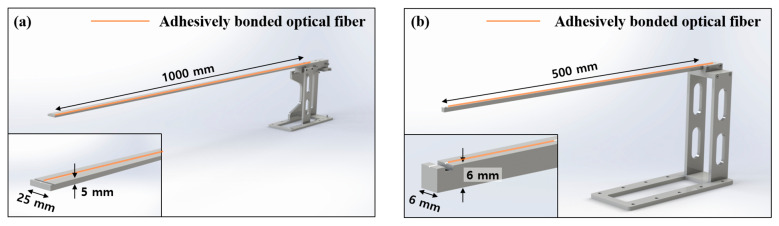
Schematic of the cantilever beams employed in the distributed strain test: (**a**) Cantilever 1 (1 m × 25 mm × 5 mm, L × W × T); (**b**) Cantilever 2 (0.5 m × 6 mm × 6 mm, L × W × T).

To verify the real-time capability of the D-OFDR system, we carried out a benchmark study on two stainless-steel cantilever beams having deliberately different dynamic characteristics. Figure 2 shows the overall configuration of Cantilevers 1 and 2. Cantilever 1 is 1 m × 25 mm × 5 mm (length × width × thickness) and is designed for a first-mode frequency close to 4 Hz; Cantilever 2 is 0.5 m × 6 mm × 6 mm, yielding a first-mode frequency near 20 Hz. Figure 2 illustrates the test arrangement and fiber layout for both specimens. For each beam, the clamped end was fixed in a rigid steel fixture while the free end projected horizontally into free space. A single-mode optical fiber was bonded along the center line of the upper surface, and an electrical resistance strain gauge (1 kHz sampling) was co-located at mid-span to provide a point reference. Free vibration was excited by imposing a small lateral tip displacement (<2% of the span) and then releasing the beam. The D-OFDR system logged full strain profiles at 100 Hz with 3 mm gauge length across the entire span, whereas the strain gauge supplied a single point history.

**Figure 3:**
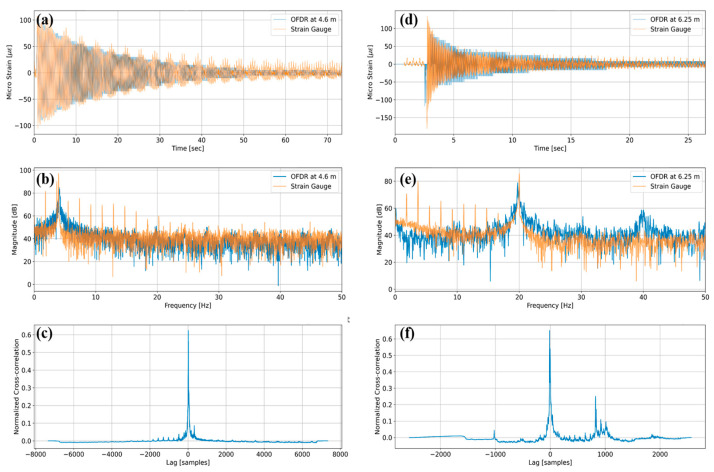
Comparison of optical and electrical measurements: (**a**) free-decay strain for Cantilever 1 at 4.6 m; (**b**) FFT amplitude map and SNR for Cantilever 1; (**c**) normalized cross-correlation between optical and gauge signals for Cantilever 1; (**d**) free-decay strain for Cantilever 2 at 6.25 m; (**e**) FFT amplitude map and SNR for Cantilever 2; (**f**) normalized cross-correlation for Cantilever 2.

Figure 3a,d show representative free-decay strain histories obtained from the 10 m-long single-mode fiber under test (FUT) bonded to two beams in line. The optical data correspond to fiber spans 4.52–5.52 m on Cantilever 1 and 6.23–6.72 m on Cantilever 2, with quantitative analysis performed at the mid-segment locations of 4.60 m and 6.25 m, respectively. Figure 3b,e show the Fast Fourier Transform (FFT) amplitude maps from the same records. For Cantilever 1, the optical channel attains the dominant frequency of 4.07 Hz compared with 3.98 Hz for the strain gauge, an error of 0.09 Hz. Notably, the strain gauge spectra in both panels include a secondary peak around 1.8 Hz, which repeats across the FFT map and is absent from the optical data. This noise is attributed primarily to periodic zero-drift compensation and low-frequency auto-balance routines in the bridge amplifier and A/D module used for the gauge channels, and it may be exacerbated by imperfect lead-wire fixation or intermittent contact; because the OFDR relies on optical frequency modulation rather than electrical bridge excitation, no corresponding comb lines appear. Figure 3c,f present the normalized cross-correlation between the time-domain strain signals obtained from OFDR and strain gauges, as shown in Figure 3a,d, yielding peak similarity values of 0.6242 and 0.6514 for Cantilevers 1 and 2, respectively, thereby confirming that the distributed sensor reproduces the temporal strain evolution of the point gauges with high fidelity.

## 3. Experimental Investigation on Reinforced Concrete Monitoring

### 3.1. Specimen Preparation and Sensor Deployment

**Figure 4:**
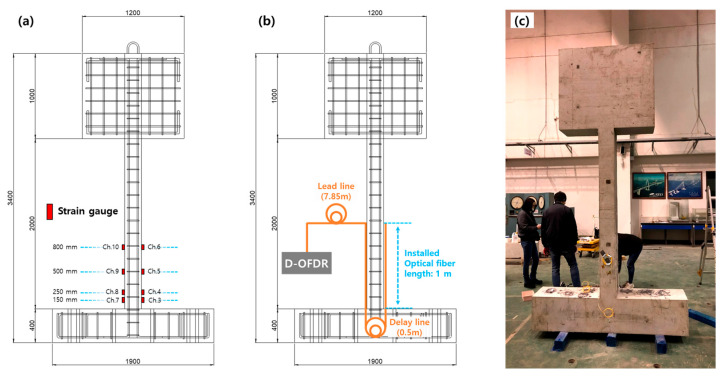
Fabrication and instrumentation of the reinforced concrete test column: (**a**) elevation drawing showing strain-gauge channels and twin fiber-optic runs; (**b**) optical routing diagram with 7.85 m lead line, 0.5 m delay loop, and 1 m bonded sensing segment; (**c**) photograph of the completed specimen mounted on the shake table with D-OFDR interface.

Figure 4 illustrates the construction of the reinforced concrete column specimen along with the deployed instrumentation network. The test article consists of a 2.0 mtall rectangular column measuring 0.40 m (width) × 0.20 m (depth) that supports a 1.0 t concrete cap, thereby imposing a constant axial load and representing an upper-story mass in a slender wall-type system. The resulting assembly takes the form of a capital letter “I”, a deliberate geometry that concentrates flexural demand in the pier portion while minimizing shear transfer through the head and footing blocks.

To measure both distributed and local strain demand, an 11 m single-mode FUT was bonded in two parallel vertical runs on the opposing faces of the column. Within this 11 m fiber, the first 7.85 m served as a transmission lead, while segments from 7.85 to 8.80 m and from 9.28 to 10.20 m corresponded to the left and right measurement regions, respectively. Additionally, eight foil strain gauges were installed at four elevations (150, 250, 500, and 800 mm above the footing), with two gauges positioned at each elevation, totaling eight gauges. The bonded fiber segments thus fully enveloped the critical plastic-hinge zone, which was expected to experience peak curvature under lateral excitation. Lead fibers were routed to the footing and connected to the D-OFDR interrogator, ensuring a protected optical path during shake-table operation.

### 3.2. Artificial Seismic-Wave Excitation Test

The instrumented RC column was rigidly anchored to a uniaxial shake table and excited by 13 sequential runs of the 1940 El Centro NS ground motion. The record was amplitude-scaled in 10% increments from 10% to 130%, producing peak spectral accelerations ranging from 0.067 g to 0.864 g—the latter corresponding to 1.6 SDS for a moderate-seismicity region. Each run had an effective duration of 40 s, and a minimum 60 s rest interval was allowed for dynamic effects to decay.

**Figure 5:**
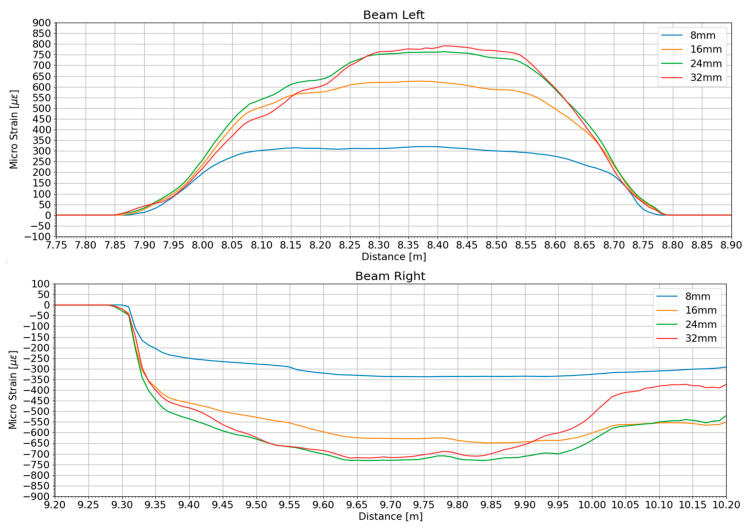
Distributed strain profiles along the left and right fiber runs for four excitation amplitudes: 10% (8 mm), 20% (16 mm), 30% (24 mm), and 40% (32 mm). Positive values denote compression on the left face, whereas negative values denote tension on the right face.

Figure 5 shows the distributed strain profiles extracted from the left- and right-side fiber runs for four representative excitation levels—10%, 20%, 30%, and 40% (denoted as 8, 16, 24, and 32 mm in the legend). On the compression face (Beam Left), strain rises sharply beyond 7.95 m, peaks near 8.35 m, and then tapers toward 8.70 m; the peak compression increases from ~350 µε at 10% to ~820 µε at 40%, indicating increasing nonlinear curvature demand. Conversely, the tension face (Beam Right) exhibits mirrored tensile strains reaching about −850 µε at 40%, with a plateau between 9.40 m and 10.00 m. The close overlap of the 24 mm and 32 mm curves on both faces suggests that localized inelasticity has developed, limiting further curvature growth despite higher input amplitude.

Overall, the distributed measurements clearly capture the emergence and growth of a localized inelastic zone, with intensifying strain localization over roughly 8.0–8.6 m (left) and 9.3–9.9 m (right) as the excitation increases. The concentration and eventual saturation of the strain peaks at higher amplitudes indicate yield penetration rather than continued elastic curvature development. These findings confirm that the D-OFDR system resolves both global softening trends and localized damage evolution under escalating seismic demands, while surpassing conventional sparse gauge arrays in spatial resolution and noise immunity. Moreover, the distributed strain profiles provide direct guidance for inspection and localized strengthening: persistent peaks and steep spatial gradients identify demand-critical regions and inform the extent and priority of targeted interventions; localized tensile spikes signal crack-prone zones and potential shear-critical regions; broadening compression-dominated zones suggest the need for added confinement to mitigate instability and surface spalling; and pronounced left–right asymmetry or migration of the strain peak with increasing input warrants checks at adjoining joints and the foundation for possible eccentric demand paths.

**Figure 6:**
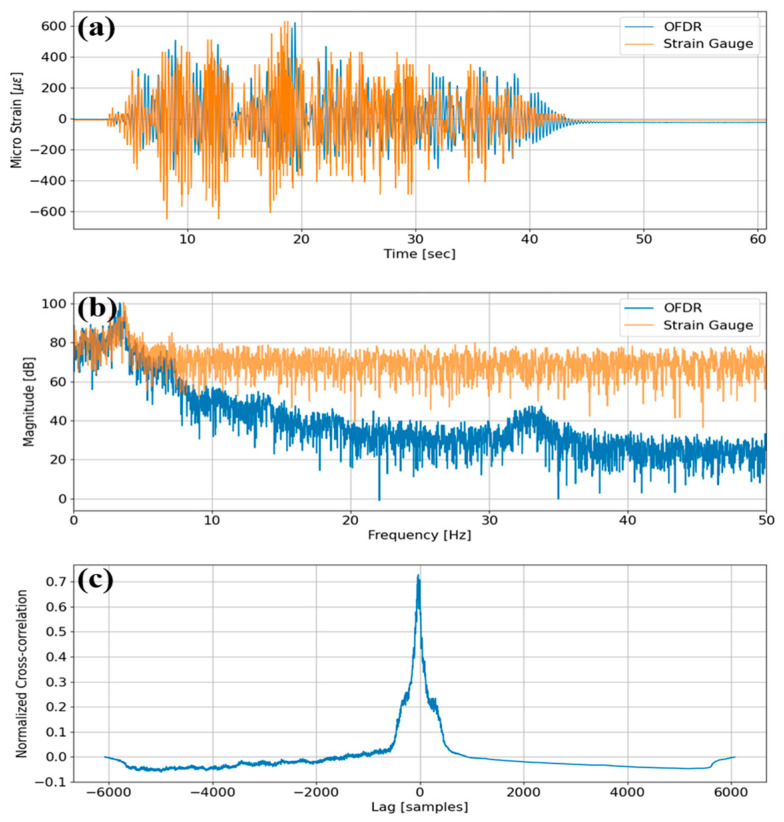
Dynamic response comparison at channel CH3 during the fourth seismic excitation step (40% PGA): (**a**) time-domain micro-strain histories measured by D-OFDR and a conventional strain gauge; (**b**) single-sided magnitude spectra derived from the same records; (**c**) normalized cross-correlation between the two signals.

Figure 6 shows three complementary diagnostics derived from the optical and electrical sensors co-located at CH3 during the 40% excitation. Figure 6a shows the time-domain micro-strain histories, which track one another closely for amplitude and decay. Figure 6b shows single-sided magnitude spectra: the optical channel achieves a peak SNR of 24.07 dB vs. 3.03 dB for the gauge, and a band-integrated SNR of 31.5 dB against 6.37 dB. The dominant frequency appears at 3.34 Hz (OFDR) and 3.60 Hz (gauge), yielding a negligible error of 0.26 Hz. Figure 6c shows the normalized cross-correlation between the time-domain strain signals from OFDR and strain gauges, visualized in Figure 6a, yielding a peak similarity of 0.6560, demonstrating high coherence in both time and frequency domains. Collectively, these metrics confirm that the D-OFDR system captures the structural response with superior signal quality and faithfully reproduces both amplitude and phase content relative to the strain gauge reference.

**Figure 7:**
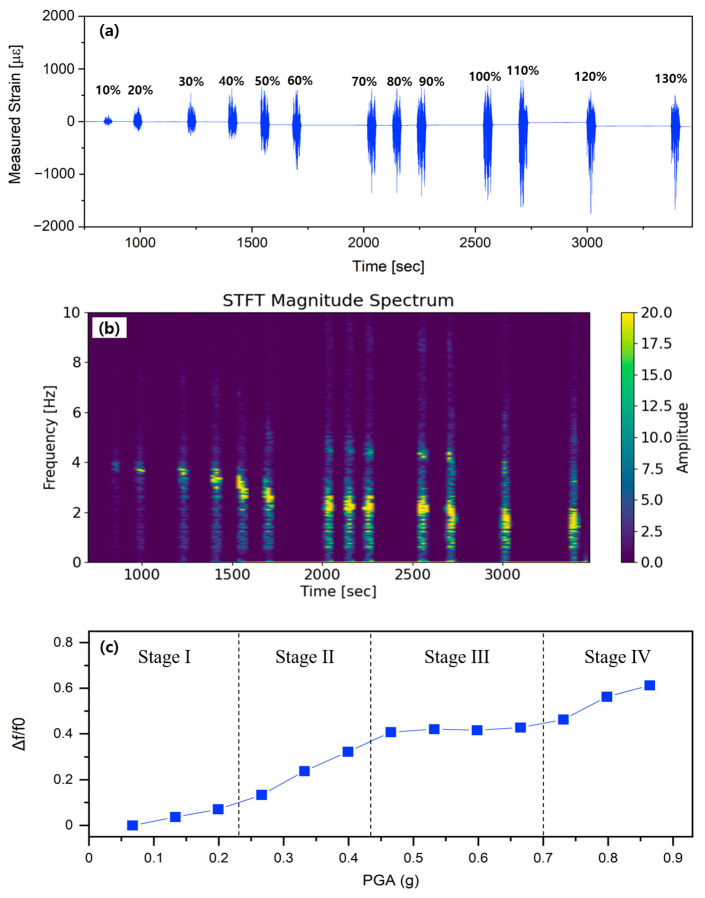
Evolution of distributed strain and resonance characteristics during thirteen incremental seismic excitations: (**a**) time-history envelopes of measured strain for input levels 10–130%; (**b**) short-time Fourier transform (STFT) magnitude spectrum revealing a progressive shift of the dominant mode; (**c**) normalized frequency shift Δf/f_0_ plotted against peak ground acceleration (PGA), quantifying stiffness degradation.

Figure 7 shows the distributed strain response captured by the D-OFDR system throughout the full excitation sequence. As evident in Figure 7a, the measured strain pulses enlarge progressively across the 13 excitations, yet the envelope ceases to increase beyond the 110% input, signaling yield-controlled stiffness. The short-time Fourier transform map in Figure 7b provides a clearer view of this degradation: the dominant frequency shifts progressively from approximately 3.82 Hz at the 10% input to approximately 1.48 Hz at the 130% run, evidencing cumulative flexural softening and stiffness loss.

Figure 7c shows the STFT-derived peak frequencies converted into the normalized shift metric Δf/f_0_ and plots them against the calculated peak ground acceleration (PGA). In Figure 7c, the horizontal axis denotes the PGA assigned to each loading step, calculated as PGA = 0.864 g × (scale/130), where 0.864 g represents 1.6 SDS, the maximum demand level of the AC-156 required response spectrum (RRS). Figure 7c then plots the normalized frequency reduction Δf/f_0_ against PGA and delineates four distinct damage regimes. Stage I (10–30%, PGA ≤ 0.26 g) constitutes the initial linear regime. The structure responds essentially elastically, and Δf/f_0_ increases only slightly—from 0.03 to 0.17—in a near-linear fashion as merely micro-level damage develops; residual capacity remains substantial and stiffness degradation is negligible. Stage II (40–60%, 0.35–0.52 g) marks the crack-initiation and damage-acceleration phase. The slope of the Δf/f_0_ curve steepens markedly, climbing to ≈0.40 as primary concrete cracks form and propagate while bond-slip emerges at the steel–concrete interface, resulting in a rapid drop in effective stiffness. Stage III (70–100%, 0.61–0.74 g) represents the crack saturation and damage stabilization phase: Δf/f_0_ levels off at 0.41 because the crack network formed in earlier stages has largely saturated, and reinforcement bridging now shares the tensile demand. Additional input therefore induces only marginal stiffness loss, and the structure enters a fatigue-stabilized regime in which damage accumulation is temporarily suppressed [20]. Stage IV (≥110%, 0.79–0.86 g) marks the yield inflection and rapid-collapse phase. Around 110% PGA the curve turns sharply upward, with Δf/f_0_ rising to 0.61 by the 130% run. This abrupt increase denotes reinforcement yielding and concrete spalling, indicating that residual stiffness is being lost at an accelerating rate; even small additional load increments produce large frequency reductions, indicating imminent soft-story collapse [21,22,23]. This staged Δf/f_0_–PGA relationship demonstrates the ability of D-OFDR to classify seismic damage progression quantitatively, offering a practical metric for real-time structural health assessment in RC members.

## 4. Discussion: Practical On-Site Application of D-OFDR

### 4.1. Real-Time Diagnostics and Post-Event Screening Scenarios

D-OFDR operates on-site in two complementary modes: in-shake, real-time diagnostics and rapid post-event screening. Typical on-site scenarios include rapid assessment of multi-story RC frames (columns, coupling beams, and shear walls) immediately after strong motion, continuous tracking of demand hotspots during aftershock sequences in high-occupancy facilities (e.g., hospitals, schools, data centers), in lifeline facilities, and in transportation infrastructure such as bridges and tunnels—including long-term condition monitoring in confined RC environments (e.g., tunnel segment rings and dam galleries) where convergence or seepage-related strains are monitored. In practice, the workflow starts with a pre-event baseline and a sensor layout aligned with expected bending axes (opposite faces per member; continuous coverage over the region of interest), followed by a short commissioning routine (see Section 4.2).

When the D-OFDR system is deployed on site, clocks are synchronized with the facility’s strong-motion reference so that the end-to-end latency (acquisition, processing, rendering) remains below one second. For seismic response of reinforced concrete members, the dominant modal content typically lies below about 20 Hz (often 0.5–10 Hz for columns and walls), so a 100 Hz update rate (a Nyquist frequency of 50 Hz) oversamples the target band by a factor of 5–100 and supports short-time spectral tracking without aliasing. In practice, analysis windows of 0.5–1.0 s provide stable Δf/f_0_ estimates while preserving responsiveness; if higher-frequency transients (e.g., bearing impact or anchor slip) are of interest, auxiliary accelerometers can be co-deployed while OFDR continues to localize distributed strain. During shaking, the system streams distributed strain to a site dashboard that renders height-wise strain heatmaps and derived cues (peak/gradient hotspots and drift proxies), enabling threshold-based actions in real time. The damage state is inferred from a joint spatial–spectral diagnostic: distributed strain profiles extracted from the bonded fibers identify peak/gradient hotspots and the contiguous length of the localized inelastic region, while a staged relative frequency-shift metric (Δf/f_0_) estimated from short-time spectra quantifies the accompanying global softening. Under shake-table excitation, the spatial localization grew and then saturated as the input amplitude increased, while the dominant frequency decreased systematically; this consistent evolution anticipated visible cracking and supports actionable thresholds for targeted inspection, localized strengthening, or temporary operational restrictions. Taken together—and supported by the installation, routing, and commissioning practices outlined in Section 4.2—these elements demonstrate that the system delivers true real-time performance and effective damage monitoring under demanding optical conditions, satisfying both the need for rapid diagnostics during shaking and practical, repeatable deployment after an event.

### 4.2. Installation, Routing, and Commissioning Guidelines

For on-site use, the interrogator is placed near the test article but outside high-vibration and dust zones, inside a sealed, IP-rated enclosure with stable power and short fiber runs to minimize loss. Sensing fibers are routed to faces where bending demand is expected to concentrate—typically along opposite vertical edges of RC columns or walls—with continuous coverage across the region of interest. To mitigate micro-bending, routes respect the manufacturer’s minimum bend radius (rule of thumb ≥ 10 × cable outer diameter, typically ≥ 30–50 mm), transition over radiused guides rather than edges, avoid point loads from tight ties by using saddle clamps or clips with compliant inserts, and include service loops of moderate diameter at equipment interfaces for strain relief. Lead cables are protected in conduit or armored sheaths, anchored at 0.3–0.5 m spacing (tightened near corners or terminations), and passed through bulkheads with grommets to prevent local pinching. Fusion splices are preferred over connectors to reduce insertion loss; where connectors are unavoidable, angled-polish terminations and careful cleaning are used to suppress back-reflection, and unused ports are capped. Bonding surfaces are lightly abraded and solvent-cleaned; fibers are attached with a thin, uniform adhesive layer to maximize strain transfer while avoiding stiff spots and trapped debris, then overcoated after cure and protected with edge guards at corners and anchor points. A short unbonded reference segment near the termination can be sleeved to isolate thermal strain over long runs. Commissioning includes continuity and insertion-loss checks, an initial OFDR baseline trace, and a light tap or controlled load to verify strain-transfer fidelity before dynamic testing. An acceptance log records the insertion-loss budget, splice count and locations, and baseline strain profiles for subsequent comparison, and all routes and terminations are labeled for maintenance. As practical targets, total added insertion loss along the sensing path should be ≤1 dB and return loss better than −35 dB, the effective frame rate ≥ 100 Hz, and the end-to-end latency < 1 s; environmental ratings should cover the site’s temperature and humidity range. Periodic rechecks of loss and baseline traces support reliable long-term operation.

### 4.3. Method Applicability and Trade-Offs for Real-Time RC Monitoring

D-OFDR is positioned as a feasible on-site method for real-time SHM of reinforced concrete structures under seismic loading, not as a mere replacement for strain gauges but as a sensing architecture with distinct capabilities and limits. Sensing-method selection in practice hinges on three axes: (i) spatial sampling (coverage and resolution), (ii) dynamic performance (update rate and end-to-end latency), and (iii) deployability and robustness under field conditions. Strain gauges provide very high bandwidth and mature calibration at a few critical points, making them valuable for reference measurements, control channels, and validation. FBG arrays multiplex many points with optical immunity to EMI and simplified cabling relative to electrical gauges; however, they still discretize space and can miss damage localized between gratings. In contrast, D-OFDR transforms a bonded fiber into a continuous strain transducer, enabling millimeter-scale spatial resolution over tens of meters with an effective data rate up to ~100 Hz. Looking ahead, advances in swept-laser coherence and noise management have the potential to extend D-OFDR to hundreds of meters with centimeter-level spatial resolution, suggesting broader applicability to long members and multi-component systems. This continuous sampling localizes inelastic regions as they form and evolve and, when paired with a staged relative frequency-shift metric (Δf/f_0_), provides a joint spatial–spectral diagnostic that anticipates visible cracking and supports actionable thresholds. For RC members, the difference between strains on opposite faces offers a practical curvature proxy that enables estimation of drift and plastic-zone length from the same distributed record. The resulting operating envelope is practical: use D-OFDR when localization of distributed responses during shaking is required; pair it with a small number of co-located gauges for calibration and fail-safe redundancy; and deploy FBG arrays where moderate spatial granularity with optical robustness and simple multiplexing is preferred. A comparative summary of the operating envelopes and trade-offs among strain gauges, FBG arrays, and the proposed D-OFDR approach is provided in Table 1.

## 5. Conclusions

In this study, we presented and validated a compact, dual-edge-triggered D-OFDR system designed for real-time structural health monitoring of reinforced concrete structures under seismic loading. The proposed D-OFDR interrogator employs a dual-interferometer architecture with a wavelength-swept laser and synchronized dual-edge triggering on both the up-chirp and down-chirp of a 50 Hz bidirectional sweep to achieve an effective 100 Hz measurement rate. Rising-edge triggers are used for up-chirps and falling-edge triggers for down-chirps, enabling Channel 1 to sample and process the up-chirp while Channel 2 simultaneously handles the down-chirp, and vice versa in the next cycle. This interleaved operation doubles the dynamic bandwidth without requiring higher performance from the laser, DAQ, or electronics. Laboratory tests on cantilever beams demonstrated that the D-OFDR system accurately measures dynamic strain and resonance frequencies with significantly enhanced dynamic signal-to-noise ratios compared to traditional point-based strain gauges. Shake-table experiments on a 2 m RC column revealed the capability of the D-OFDR system to reliably monitor progressive structural degradation under incremental seismic loading. The system effectively identified distinct damage stages through distributed strain measurements and frequency-domain analyses, with the fundamental resonant frequency decreasing notably from approximately 3.82 Hz at initial loading levels (10% PGA) to approximately 1.48 Hz at maximum loading (130% PGA). These findings highlight the advantage of D-OFDR in detecting subtle yet critical structural integrity changes, providing valuable insights that go beyond conventional strain monitoring. Overall, the D-OFDR system demonstrated superior spatial coverage, robustness against electromagnetic interference, and sensitivity to structural damage, substantially outperforming conventional point sensors.

Importantly, the demonstrated system also fills critical gaps in current seismic monitoring technologies. Unlike point sensors and sparse FBG arrays that leave unmonitored spans, the distributed OFDR measurements provide continuous, millimeter-scale coverage that resolves the emergence of a critical inelastic region and yield penetration prior to visible cracking. The dual-edge interleaving enables practical, in-shake update rates (100 Hz), overcoming the refresh-rate limitations that often prevent real-time tracking of modal shifts and stiffness loss. The optical front end improves dynamic SNR while remaining inherently immune to EMI, reducing noise pathways that compromise electrical gauges during strong motion. Moreover, linking the distributed strain-profiling results with a staged frequency-shift metric (Δf/f_0_) produces a joint spatial–temporal diagnostic: the profiles localize where inelastic demand concentrates and how it grows along the member, while Δf/f_0_ quantifies the accompanying global stiffness reduction. This coupling enables component-specific, real-time decision thresholds—ranging from targeted inspection and localized repair to retrofit initiation and temporary shutdown—that conventional strong-motion stations, post-event visual inspections, and sparse FBG arrays cannot supply. In this way, the system addresses key shortcomings in current practice: lack of continuous spatial coverage, absence of actionable update rates during shaking, and absence of calibrated, strain-based damage staging directly tied to distributed measurement.

Future work will further extend this monitoring approach to larger-scale structures, enhance data-processing methodologies, and refine the system for long-term deployment in practical structural health monitoring applications.

## Figures and Tables

**Table 1:** Comparative operating envelopes and trade-offs of strain gauges, FBG arrays, and Rayleigh-OFDR for on-site SHM of reinforced concrete under seismic loading.

Attribute	Strain Gauge (Electrical)	FBG Array (Point-Multiplexed Optics)	D-OFDR (This Work)
Spatial sampling	Point only; gaps between points	Discrete points (cm–m spacing)	Continuous along fiber; ~mm over tens of m
Real-time dynamics (seismic band)	Very high at instrumented points	High-rate per channel (demodulator-dependent)	~100 Hz distributed; <1 s end-to-end
EMI/noise	EMI/corrosion/wiring sensitive	EMI-immune	EMI-immune; high dynamic SNR
Install & maintenance	Many wires; shielding; periodic replacement	Good bonding/anchoring and surface prep; fewer cables via multiplexing; connector hygiene; temperature compensation	Good bonding/bend-radius control; low-loss routing; baseline/latency management
RC-specific diagnostics	Curvature/drift only where paired gauges exist; no continuous zone length	Curvature/drift possible at grating pairs; may miss between points	Opposite-face curvature/drift from continuous profiles; inelastic-zone length; staged Δf/f_0_ for global softening
Best use-cases	Calibration/validation; control points; local high-rate events	Multi-point with moderate granularity	In-shake localization; post-event screening; component-level triage
Key limitations	Un-instrumented spans; wiring complexity; durability	Still discrete; may miss between gratings; per-point cost	Shorter range than low-rate DFOS; sensitive to adhesive/micro-bending; careful installation

## Data Availability

Data are contained within the article.
